# Meat Proteins as Dipeptidyl Peptidase IV Inhibitors and Glucose Uptake Stimulating Peptides for the Management of a Type 2 Diabetes Mellitus In Silico Study

**DOI:** 10.3390/nu11102537

**Published:** 2019-10-21

**Authors:** Paulina Kęska, Joanna Stadnik, Olga Bąk, Piotr Borowski

**Affiliations:** 1 Department of Animal Raw Materials Technology, Faculty of Food Science and Biotechnology, University of Life Sciences in Lublin, Skromna 8, 20-704 Lublin, Poland; paulina.keska@up.lublin.pl; 2 Faculty of Chemistry, Marie Curie-Sklodowska University in Lublin, 3 Marie Curie-Sklodowska Sq., 20-031 Lublin, Poland; olga.bak@poczta.umcs.lublin.pl (O.B.); piotr.borowski@poczta.umcs.lublin.pl (P.B.)

**Keywords:** bioactive peptides, DPP-IV inhibitors, in silico, pork proteins

## Abstract

Diabetes mellitus is a non-communicable disease entity currently constituting one of the most significant health problems. The development of effective therapeutic strategies for the prevention and/or treatment of diabetes mellitus based on the selection of methods to restore and maintain blood glucose homeostasis is still in progress. Among the different courses of action, inhibition of dipeptidyl peptidase IV (DPP-IV) can improve blood glucose control in diabetic patients. Pharmacological therapy offering synthetic drugs is commonly used. In addition to medication, dietary intervention may be effective in combating metabolic disturbances caused by diabetes mellitus. Food proteins as a source of biologically active sequences are a potential source of anti-diabetic peptides (DPP-IV inhibitors and glucose uptake stimulating peptides). This study showed that in silico pork meat proteins digested with gastrointestinal enzymes are a potential source of bioactive peptides with a high potential to control blood glucose levels in patients with type 2 diabetes mellitus. Analysis revealed that the sequences released during in silico digestion were small dipeptides (with an average weight of 270.07 g mol^−1^), and most were poorly soluble in water. The selected electron properties of the peptides with the highest bioactivity index (i.e., GF, MW, MF, PF, PW) were described using the DFT method. The contribution of hydrophobic amino acids, in particular Phe and Trp, in forming the anti-diabetic properties of peptides released from pork meat was emphasized.

## 1. Introduction

In recent years, the rate of diabetes mellitus morbidity has systematically grown, becoming one of the most common health problems among people around the world. It is estimated that in the past thirty years, the percentage of patients has increased almost four-fold, reaching approximately 422 million adults with diabetes mellitus in 2014 (for comparison, 108 million people were reported as having diabetes mellitus in 1980) [1]. Diabetes mellitus is a serious chronic disease associated with abnormal blood glucose levels in the body. It belongs to the group of non-communicable diseases (NCDs) caused by metabolic disorders [1]. Type 1 diabetes mellitus (T1DM) and type 2 diabetes mellitus (T2DM) are the main types of diabetes, wherein T2DM is much more common and represents 90%–95% of all diabetes cases [1,2]. The causes of the epidemic of T2DM are embedded in the very complex relationship between genetic systems (e.g., ethnic origin, age) and environmental factors (e.g., unhealthy diet, sedentary lifestyle leading to the growth of obesity among the population). Some of them may be modified behaviorally, for example, by practicing a healthy lifestyle and adoption of an appropriate diet, thus prevention and treatment of diabetes mellitus can be effectively implemented. Another therapeutic strategy is the inhibition of the metabolic enzymes involved in the regulation of blood glucose levels, e.g., alpha-amylase and alpha-glucosidase (carbohydrate-cleaving binding—the inhibition of the enzyme causes a delay in the absorption of carbohydrates in the gastrointestinal tract) or dipeptidyl peptidase IV (DPP-IV) (hydrolyzing peptide bonds—the inhibition of the enzyme increases the half-life of substances affecting insulin levels) [2,3].

**Figure 1:**
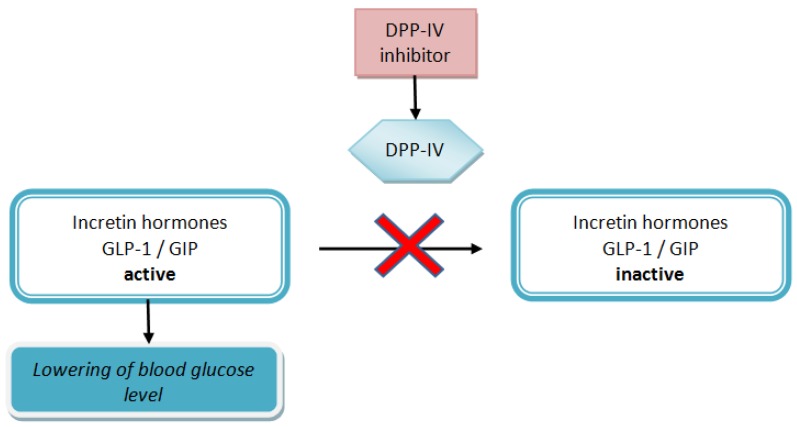
The scheme of the activity of the dipeptidyl peptidase IV (DPP-IV) inhibitor; GLP-1: glucagon-like peptide-1; GIP: glucose-dependent insulinotropic peptide.

Incretins (intestinal insulinotropic hormones) are primarily responsible for regulating the level of glucose. Two peptide incretin hormones involved in the control of blood glucose have been identified in humans, namely, glucose-dependent insulinotropic peptide (GIP) and glucagon-like peptide-1 (GLP-1) [4,5]. They are released from the gut in response to food intake and exert a potent insulinotropic effect helping to maintain control of postprandial glucose levels. However, the incretins are rapidly inactivated by the enzyme DPP-IV (Figure 1). Dipeptidyl peptidase IV, also known as adenosine deaminase binding protein or CD26 (EC 3.4.14.5), is a dimeric (2 × 110 kDa subunits) serine aminopeptidase. It catalyzes the release of X-Pro or X-Ala (wherein X is any amino acid) fragments from the N-terminus of peptides [6].

The medical strategy in the fight against disorders causing diabetes is to block the function of DPP-IV. Synthetic inhibitors of DPP-IV are used to increase the half-life of active GLP-1 and GIP, usually in the pharmacological approach to treatment, leading to a significant increase in their concentration in the blood. In turn, the GLP-1 and GIP contribute to lower glucose levels by stimulating insulin secretion and inhibiting the release of glucagon, enabling effective efforts against hyperglycemia [2,7].

In addition to pharmacological treatment, natural sources of bioactive compounds with anti-diabetic activity are sought [8]. One of the probable mechanisms of action of anti-diabetic agents from food sources may result their ability to inhibit the DPP-IV enzyme. Lacroix and Li-Chan [9], in their in silico study, demonstrated that raw materials of animal origin are better sources of DPP-IV inhibitors as compared to plant sources. As an example, peptides released from cow’s milk, beef, poultry, fish, eggs, and aged meat products have been proposed as a source of potential inhibitors of DPP-4 [2,9,10].

The data available in the literature show that pork meat can be a source of bioactive peptides which appear to have the potential to promote health benefits for consumers. When they are released from parent sequences and absorbed into the blood from the gastrointestinal lumen, they can influence the functioning of the body, i.e., cardiovascular system, digestive, hormonal, immune, and nervous systems [11]. They may also be effective in the prevention of the need for and/or assist in the treatment of T2DM, and they may become an important therapeutic strategy [12]. The bioinformatic approach to the discovery of bioactive peptides enables the characterization of peptides produced in terms of their theoretical physico-chemical, bioactive properties [13,14] and sensory properties [15]. Thus, information obtained from in silico analyses may help food technologists to take steps to remove or limit the production of allergenic, toxic or bitter peptides while maintaining the desired bioactivity. As an example, in silico and in vitro approaches were combined to determine the potency of antimicrobial peptides derived from porcine proteins (*Sus scrofa*) and beef proteins (*Bos taurus*) isolated from dry-cured meat products [16]. Also, the allergen potential of peptides isolated from beef fermented with acid whey after 31 days of ripening was determined using in silico methods [17]. In other studies, the in vitro and in silico approach were combined for determination of the potential of pork peptides and proteins as antioxidants [13] and angiotensin-converting enzyme inhibitors [14] obtained from dry-cured pork loins after in vitro digestion with gastrointestinal enzymes. Recently Sayd et al. [18] used combined in vivo and in silico approaches for predicting the bioactive peptides from meat digestion and confirmed the presence of biologically active sequences after the digestion of cooked beef in the digestive tract of mini pigs fitted with gastric cannulas received. Gallego, Aristoy, and Toldrá [10] also evaluated the potential of DPP-IV inhibitory peptides which may be present in the water-soluble extract of dry-cured Spanish ham. The bioactivity of the identified peptides by the mentioned authors has been confirmed by chemical synthesis and in vitro assays showing that KA and AAATP peptides have the strongest DPP-IV inhibitory activity (IC_50_ values of 6.27 mM and 6.47 mM, respectively). The AA, GP, PL, and carnosine dipeptides as well as AAAAG, ALGGA, and LVSGM peptides were also DPP-IV inhibitors, although to a lesser extent. These findings confirm the potential of meat products as a natural precursor of DPP-IV inhibitory peptides.

It is thought that the amino acids and short peptides supplied with the diet may have anti-diabetic activity in many ways. In addition to the inhibiting properties of the peptides, these activities were distinguished by direct stimulation of pancreatic cells leading to increased insulin secretion and the effect on the secretion of hormones from the incretin group [19,20]. The biological activity of the peptides may also result from their physico-chemical characteristics deriving from the amino acid composition and their arrangement in the peptide chain. Most of the studies conducted to date focused on the production and identification of DPP-IV inhibitory peptides from protein hydrolysates from the food system. To realize the potential of proteins from pork meat, more information is needed regarding the nature of the physical and chemical properties of the peptide sequences that can modulate blood glucose levels in the body. This knowledge can be used in the design of an appropriate diet therapy in order to reduce NCDs.

Significant progress has been made in the design of structure-based drugs at various stages of drug discovery. In silico methods based on molecular and quantum mechanics, such as docking, molecular dynamics, as well as ab initio and density functional theory (DFT) chemical reactivity calculations, bring us closer to understanding the metabolism of drugs and predicting the interaction among drugs [21]. Pharmacophore modeling is a widely utilized method in the computer-aided drug design process. Also, the application of computational methods in the determination of the relationship between the structure and biological activity of the molecule is widely used. The quantitative structure–activity relationship (QSAR), providing the quantitative relationship between the structure of a compound and its action, is based on the assumption that biological properties of molecules are mathematical functions of their physico-chemical parameters (descriptors). In turn, quantitative structure–property relationship methods (QSPR) allow to determine the qualitative relationship between the structure of a compound and its physical properties. In order to determine the structure–activity relationship, several groups of descriptors, such as structural and topological, electronic (including the highest occupied and lowest unoccupied molecular orbitals—HOMO and LUMO, respectively), geometric, and thermodynamic, are selected [22]. In the QSAR model, the DFT is useful in the prediction of biological activity or toxicity of molecules. Density functional theory is a quantum chemical method useful for predicting the properties of molecules with rather low computational effort on the basis of their electron density. The DFT results were used in the QSAR analysis, among others, for the evaluation of antibacterial activities against Gram-positive (*Staphylococcus aureus*) and Gram-negative bacteria (*Klebsiella pneumonia*, *Proteus bacilli*, and *Shigella flexneri*) of quinazolinone compounds [23]. In turn, Kuruvilla et al. [24] calculated, inter alia, the HOMO and LUMO energies using the DFT method, and the obtained results were used in the QSAR analysis to understand the stability, reactivity, and bioactivity of the test compound (4-[2-(Dipropylamino) ethyl]-1,3-dihydro-2H-indol-2-one).

The aim of this study was to determine the in silico potential of sixteen selected pork muscle proteins (*Sus scrofa*) to generate bioactive peptides with anti-diabetic properties. At first, evaluation of the potency of the intact porcine proteins as sources of bioactive peptides was carried out, and, next, the prediction of the bioactive potential of peptide sequences released after the simulated digestion was made. Finally, the five peptides with the highest bioactivity potential were selected and their electronic properties (orbital energy values: HOMO and LUMO) were calculated by density functional theory.

## 2. Materials and Methods

Sixteen protein sequences from porcine (*Sus scrofa*) skeletal muscle were analyzed (Table 1). All sequences were derived from UniProtKB database [25]. The profiles of the potential biological activity of proteins for generating bioactive peptides by using “*Profiles of potential biological activity*” tools available in the BIOPEP-UWM database were determined [26]. The value of selected proteins as precursors of anti-diabetic bioactive peptides, based on the frequency of bioactive fragments of the tested activity in the protein chain (parameter A) and peptide affinity for the specific receptor characterizing the potential activity (parameter B) (mM^−1^) was evaluated [27].

The calculations were made using the “*Calculation*” tools available in the BIOPEP-UWM database. The analyzed proteins were also subjected to in silico proteolytic digestion by the digestive enzymes pepsin (EC 3.4.23.1); trypsin (EC 3.4.21.4); and chymotrypsin (EC 3.4.21.1) (“*Enzymes action*” tools available in the BIOPEP-UWM database). The frequency of the release of fragments with a given activity by selected enzymes (parameter A_E_) and the relative frequency of release of fragments with given activity by selected enzymes (parameter W) was determined [11,27,28]. The peptide fragments obtained from the in silico digestion were analyzed for their physico-chemical properties using bioinformatics tools. The molecular weight, isoelectric point, charge, and solubility of potential anti-diabetic peptides was estimated using the PepStat tools [29]. The potential general bioactivity of the peptides obtained in this study were confirmed by PeptideRanker [30].

The quantum chemical calculations were carried out in the following way. Equilibrium geometries and harmonic vibrational frequencies of the selected peptides GF, MW, MF, PF, and PW were determined at the B3LYP [31] level with the 6-311++G** basis set [32,33]. All optimized structures were confirmed to be local minima (all harmonic frequencies turned out to be real). Calculations were performed using the PQS quantum chemistry package [34,35]. It was decided to do all calculations with tightened integral prescreening thresholds (by two orders of magnitude), and a high-quality integration grid was used in the self-consistent field (SCF) procedure.

The Pearson correlation coefficient was computed by means statistics program (Excel; Microsoft) for determining the relationship between the physico-chemical parameters and general bioactivity score.

## 3. Results and Discussion

The analysis of the potential biological activity profile showed that the proteins of *Sus scrofa* contained in their sequence’s numerous bioactive peptides effective against diabetes mellitus. All the selected proteins—eight myofibrillar and eight sarcoplasmic, presented in Table 1—proved to be a potential source of DPP-IV inhibitors which represented more than half of all of bioactive fragments. Research conducted by Kęska and Stadnik [36] indicated that myofibrillar proteins are a more abundant source of biologically active fragments (6330 sequences) compared to the sarcoplasmic proteins (3534 sequences). As shown in this study, the percentage of DPP-IV inhibitors in the total volume of biologically active fragments was similar among groups of proteins selected for analysis, i.e., 50.51% for the sarcoplasmic proteins and 52.91% for the myofibrillar proteins. Also, the assessment of pork meat proteins as precursors of peptides with angiotensin I-converting enzyme inhibitory properties showed that the percentage of the bioactive peptides in general does not depend on the protein fractions and reaches about 31.64% in each of them [36]. As observed in this study, porcine muscle proteins are also a source of regulating glucose level peptides (glucose uptake stimulating peptide, GUSP), which showed a different tendency. Almost two-fold more of these peptides were obtained from sarcoplasmic proteins (2.94%) than from myofibrillar proteins (1.89%).

The parameter A (Table 2) was used as the quantitative measure of porcine meat proteins as precursors of biologically active peptides having an activity of DPP-IV and GUSP. Guided by the principle, the higher the index value, the richer the source of a sequence with a given activity, TTN (out of myofibrillar proteins; 0.6713) and GAPDH (of the sarcoplasmic proteins; 0.6697) were distinguished as the best precursors of peptides inhibiting DPP-IV. The latter of the abovementioned proteins are also characterized by a high (but not the highest) value of the parameter B, determining the affinity of the peptide to a specific receptor characterizing its potential biological activity. Moreover, TNNT1, TNNT3, and MB proved to be good sources of GUSP (parameter A was 0.1489, 0.1218, and 0.0714, respectively, Table 2).

The in silico hydrolysis of the selected protein sequences by enzymes of the gastrointestinal tract (pepsin, trypsin, and chymotrypsin) was performed, and the results of the in silico proteolysis are shown in Table 3. The resulting peptide fragments showed resistance to digestion in the gastrointestinal tract, confirming the high potential of pork meat proteins to generate the bioactive peptides after intake of a meal. The digestive enzymes were most effective in the release of DPP-IV inhibitory sequence from MYH2 and PYGME, reflected by the high value of A_E_ and W. On the other hand, TNNT1 and ALDOA were the most effective in the release of GUSP under the action of digestive enzymes (Table 3).

Fifty-four different peptide sequences released by in silico hydrolysis were listed and analyzed by bioinformatics tools (Table 4). Undertaken analyses showed the multifunctionality of individual peptides—the sequences having glucose uptake (GUSP) properties were also inhibitors of DPP-IV. The obtained sequences were mostly dipeptides (98%) with an average weight of 270.07 g mol^−1^ (±36.71 g mol^−1^). The literature data suggest a role of short sequences derived from food proteins in prevention of diabetes mellitus, which indicated a greater role of dipeptides compared to the three amino acid sequences or of free amino acids in the inhibition of DPP-IV [37,38]. Short peptide fragments are also able to be absorbed easier from the gastrointestinal tract into the blood [39] and then may cause a physiological effect on the human body. As a result of in silico digestion, only one triple amino acid sequence, VPL, was released by digestive enzymes from NEB (Table 4). This tripeptide is used as a synthetic DPP-IV inhibitor effective in inhibiting the degradation of GLP-1, known under the name Diprotin B [4,40]. Food-derived proteins are also the source of other peptide fractions, which were used in pharmacology as a synthetic equivalent. Another tripeptide, IPI (also known as Diprotin A), which is encrypted in the κ-casein sequence, is the most potent (IC_50_ = ~4 µM) of the presently known peptides inhibiting DPP-IV [41].

Over half of the analyzed sequences had an isoelectric point (pI) of approximately 3.5. The pI is defined as the pH at which the protein load is 0. This is a property that influences the maintenance of the biological activity of the peptides in specific pH conditions. Sequences with pI = 3.5 simultaneously have a net charge value equal to 0 and exhibit poor solubility in water. Eight sequences were characterized by different properties (pI of about 10.45; net charge equal to 1, good solubility in water) (Table 5).

Nongonierma et al. [42] analyzed the relationship between physico-chemical properties of peptides (including length, isoelectric point, hydrophobicity, and net charge value) with the biological activity of peptides. This approach was also presented in this study. An analysis of the Pearson’s correlations among the selected physico-chemical properties of peptides and their bioactivity was performed based on the data presented in Table 5. The majority of parameters obtained a weak or moderate correlation coefficient (*r* = in the range from 0.010 to 0.573), except for the isoelectric point and net charge for which a strongly positive correlation was observed (*r* = 0.907).

**Figure 2:**
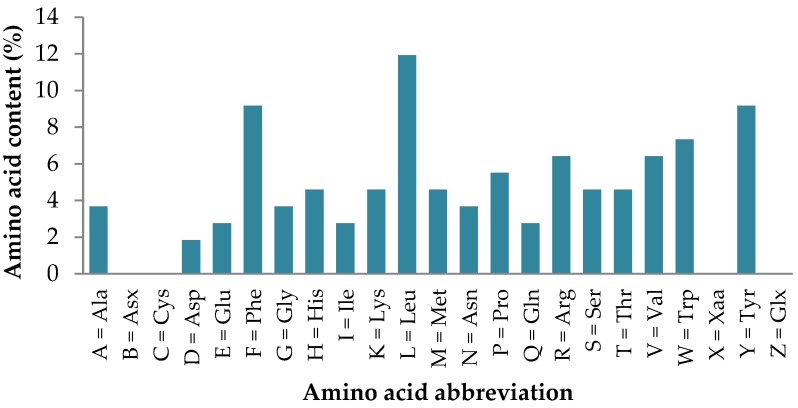
Percentage share of amino acids present in the sequences of bioactive peptides inhibiting the activity of the DPP-IV enzyme.

There are many examples of biologically active food proteins, exhibiting the physiological role in addition to the dietary requirements. Underlying these activities, apart from the physico-chemical properties, is the relationship between structures and their function. In the case of peptides derived from food proteins involved in the regulation of blood glucose levels, there is not enough yet understood. Considering the above, the analyzed sequences were subjected to further parametric evaluation assessing the overall bioactive potential of received sequences using PeptideRanker software. Of the 54 peptide fragments, 13 were characterized by high bioactivity (a value above 0.93); they were: AF; AW; GF; HF; IW; MF; MW; NF; PF; PW; SF; SW; and QF (Table 5). Glucose regulation by specific amino acids could prove to be an important non-insulin dependent mechanism for glucose control in insulin-resistant individuals, such as those with T2DM. In the present study, it was observed that two hydrophobic aromatic amino acids (i.e., Phe or Trp) exist in each of the specified sequences. The results suggest the contribution of hydrophobic amino acids to the specific properties of bioactive sequences involved in the management of anti-diabetic proteins, which corresponds to other research [9,20,38,43]. Research carried out by Nongonierma and Fitzgerald [20] showed that the hydrophobic amino acids located at the N-terminus of the peptides have a tendency to decrease the IC_50_ value of DPP-IV inhibitor (the lower IC_50_ value means the higher activity of the peptide). Analysis of peptides conducted by Lan et al. [38] and Tulipano et al. [43] revealed the influence of the presence of Trp at the N-terminus of the peptide on their DPP-IV inhibitory properties. Therefore, the specific properties of the peptide may be a consequence of their amino acid composition. For this reason, an analysis of the percentage of individual amino acids forming peptides (54 sequences) was made, and the results are shown in Figure 2.

The analyzed sequences consisted of 19 amino acids, of which Leu (11.93%), Phe (9.17%), Tyr (9.17%), and Trp (7.34%) accounted for the largest share. According to other authors [38,44,45], some peptides (i.e., Trp-Val, Phe-Leu, His-Leu; Leu-Leu, Val-Val, and Trp-Arg) are potent inhibitors of DPP-IV. Recent evidence suggests that the amino acid Leu may also influence glucose sensing pathways in the hypothalamus thereby regulating whole-body glucose and energy metabolism in ways that are not currently well understood [46]. Amino acid composition may also affect the mode of action of the biologically active peptide to specific receptors on target cells. Other studies reported that the peptide inhibitors of DPP-IV containing Pro have a high activity [47]. The inhibitory effects of the peptides may result from different modes of action and inhibition of the enzyme. Peptides derived from food sources containing Pro at the P1 position can act as a substrate for the enzyme (competitive mode of action). On the other hand, most of the peptides containing Trp at the N-terminus of the peptide display uncompetitive or non-competitive inhibition against DPP-IV [41]. Peptides can stimulate glucose uptake in skeletal muscles through other molecular pathways independently of insulin or by increasing insulin sensitivity in target cells, resulting in increased glycogen contents in skeletal muscle. Dipeptides containing branched-chain amino acids, such as Ile-Leu and Val-Leu, have been reported to stimulate glucose uptake in skeletal muscles possibly via kinase signaling pathways, which are different from the mechanism of the insulin-stimulated glucose transporters [48]. Furthermore, dipeptides Ile-Leu and Val-Leu could exert positive effects on glucose regulation by both DPP IV inhibition and stimulating glucose uptake activity (Table 4).

In further analysis, the five peptides with the highest bioactivity index, from 0.99 to 1.00 (GF, MW, MF, PF, PW, Table 5) were selected and their selected electron properties using the DFT method were described. This approach may be helpful in developing drugs based on peptides, and the values determined in this study may serve as descriptors to determine the relationship between structure and activity of a biological compound/drug. The DFT method is applied to study the structural, electronic, and dynamic properties of a molecule and plays a vital role in the drug discovery process.

Density functional theory was used to find the values of orbital energy from which we can predict how well a group can pass or accept electrons. This study is possible by calculating the HOMO and LUMO energies. This approach was used, inter alia, for identification of important chemical features of 11β-Hydroxysteroid dehydrogenase type1 inhibitors which are involved in the metabolism of glucose [49]. An important parameter of the molecular electron structure is the frontier orbital, HOMO. A higher HOMO value implies that the molecule has good electron donating ability. The structure and calculated HOMO and LUMO values are summarized in Table 6.

**Figure 3:**
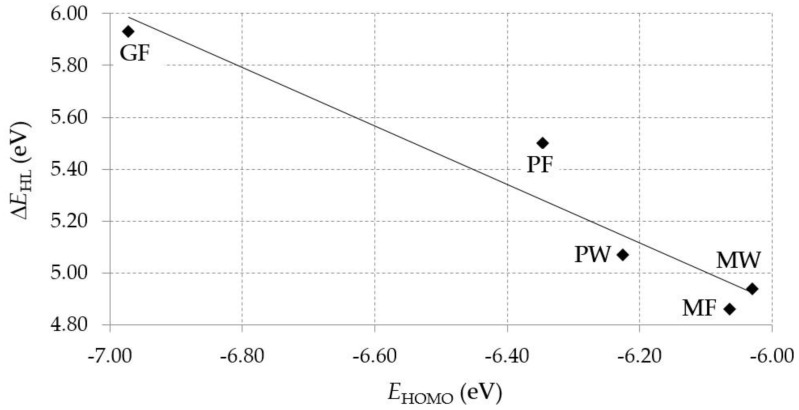
Relationship between the energy gap Δ*E*_HL_ and the HOMO orbital energy *E*_HOMO_ of selected peptides from *Sus scrofa*.

The biological activity of a compound is closely related to its electronic structure. The importance of HOMO and LUMO with regard to that issue has been emphasized many times by several authors [24,49,50]. In particular, the HOMO orbital energy, *E*_HOMO_, can be correlated with the electron-donating character, in that the higher (less negative) the energy, the more electron donating ability of the molecule. Similarly, the lowering of the HOMO–LUMO energy gap, Δ*E*_HL_, indicates the increasing role of the charge transfer interactions within the molecule [51]. Therefore, we carried out the DFT calculations to visualize more clearly the correlation between the biological activity and the electronic structure of the five considered dipeptides: GF, MW, MF, PF, and PW. The contours of the HOMO and LUMO orbitals are reported in Table 6. In addition, their orbital energies and Δ*E*_HL_ energy gaps are also given. A clear trend of a decreasing Δ*E*_HL_ value along with an *E*_HOMO_ increase was observed, see Figure 3. Obviously, dipeptides located at the bottom-right corner of the figure are predicted to exhibit the highest biological activity. Indeed, the bioactivity score of MW and MF, being equal to 1, turned out to be the highest among all investigated dipeptides. On the other hand, PF and GF, exhibiting lower HOMO orbital energy, showed slightly worse bioactivity.

Establishing the health effects of bioactive peptides from meat is an active area of clinical research. Recently Montoro-García et al. [52] showed that the consumption of 80 g/day dry-cured ham did not affect sodium excretion nor blood pressure. Consuming dry-cured ham with bioactive peptides, among other bioactive compounds, has been proven to improve lipid and glucose metabolism in humans. However, additional studies are needed to confirm the effects of meat bioactive peptides on diverse risk factors in pathological conditions, which is also the case with the results of the present study.

## 4. Conclusions

Bioactive peptides derived from food proteins may cause certain physiological reactions in the body, which has a beneficial effect on health. These peptides are attractive to researchers and consumers because of their potential for use in functional foods and other interventions or control of lifestyle-related diseases. However, the cost-effectiveness of the peptides used in the design of health-promoting agents is substantially limited, inter alia, by balancing the high efficiency of obtention while retaining the biological activity of the active ingredients of the food. To address these challenges, we proposed the use of an in silico approach as useful tool. Complementary empirical methods are useful to assess the potential of proteins as precursors of bioactive peptides. Therefore, the objective of this study was to consolidate all findings to date on the peptide sequences, which are presented as biologically active using an in silico approach. The study showed that pork meat proteins are a potential source of bioactive peptides. Digested in silico by gastrointestinal enzymes, they have a high potential for the management of blood glucose levels in patients with T2DM. The inhibitory activity of the peptides against DPP-V accounted for about 50% of the total active peptides recorded in the protein sequences. The database of proteins and bioactive peptides—BIOPEP-UWM—is a useful tool for the analysis of anti-diabetic peptides derived from the precursor proteins in the in silico model studies. It enables quick and easy assessment of the potential of pork meat proteins as major functional components of meat products important in the prevention and assistance of treatment for T2DM. In the future, the accumulated knowledge will also the analysis of large datasets on proteins and peptides and the understanding of interactions with biological targets (specific receptors) and structure–activity relationships.

## Figures and Tables

**Table 1:** The porcine proteins subjected to in silico analysis.

Protein Name	Abbreviation	Entry Name (ID)	Protein Name	Abbreviation	Entry Name (ID)
Myofibrillar Proteins			Sarcoplasmic Proteins		
Actin, alpha skeletal muscle	ACTS	P68137	Myoglobin	MB	P02189
Myosin-2	MYH2	Q9TV63	Creatine kinase U-type,	CKMT1	Q29577
Tropomyosin alpha-3 chain	TPM3	A1XQV4	Creatine kinase M-type	CKM	Q5XLD3
Troponin C, skeletal muscle	TNNC2	P02587	Glyceraldehyde-3-phosphat dehydrogenase	GAPDH	P00355
Troponin T, fast skeletal muscle	TNNT3	Q75NG9	L-lactate dehydrogenase A-chain	LDHA	P00339
Troponin T, slow skeletal muscle	TNNT1	Q75ZZ6	Phosphoglycerate kinase 1	PGK1	Q7SIB7
Titin (fragment)	TTN	Q29117	Alpha-1,4 glucan phosphorylase	PYGM	F1RQQ8
Nebulin	NEB	Q3Y5G4	Fructose-bisphosphate aldolase	ALDOA	Q6UV40

**Table 2:** Results of the potency evaluation of the intact porcine proteins as sources of bioactive peptides.

Protein	DPP-IV Inhibitory		Stimulating ^a^	
	Parameter A	Parameter B	Parameter A	Parameter B
ACTS	0.6499	0.000258	0.0451	-
MYH2	0.5910	0.000255	0.0552	-
TPM3	0.5035	0.000141	0.0810	-
TNNC2	0.5031	0.000247	0.0181	-
TNNT3	0.5941	0.000237	0.1218	-
TNNT1	0.5196	0.000323	0.1489	-
TTN	0.6713	0.000307	0.0385	-
NEB	0.6475	0.000361	0.0296	-
MB	0.6494	0.000534	0.0714	-
CKMT1	0.6394	0.000200	0.0457	-
CKM	0.6352	0.000434	0.0472	-
GAPDH	0.6697	0.000401	0.0300	-
LDHA	0.6175	0.000341	0.0542	-
PGK1	0.6451	0.000395	0.0432	-
PYGM	0.6449	0.000413	0.0356	-
ALDOA	0.6147	0.000659	0.0642	-

^a^ Glucose uptake stimulating peptide. Parameter A—the frequency of bioactive fragments occurring in a protein sequence. Parameter B—the potential biological activity of a protein.

**Table 3:** Frequency of the release of fragments with a given activity by selected enzymes (parameter A_E_) and the relative frequency of release of fragments with given activity by selected enzymes (parameter W) from porcine proteins after in silico digestion.

Protein	DPP-IV Inhibitory		Stimulating ^a^	
	Parameter A_E_	Parameter W	Parameter A_E_	Parameter W
ACTS	0.0307	0.0496	0.0026	0.0598
MYH2	0.0414	0.0737	0.0030	0.0567
TPM3	0.0272	0.5670	0.0034	0.0455
TNNC2	0.0182	0.0380	-	-
TNNT3	0.0249	0.0440	0.0036	0.0307
TNNT1	0.0257	0.0492	0.0074	0.0516
TTN	0.0270	0.0423	0.0017	0.0457
NEB	0.0371	0.0604	0.0017	0.0607
MB	0.0437	0.0706	0.0063	0.0916
CKMT1	0.0465	0.0766	0.0070	0.1584
CKM	0.0456	0.0760	0.0051	0.1118
GAPDH	0.0232	0.0584	-	-
LDHA	0.0320	0.0548	0.0029	0.0587
PGK1	0.0348	0.0568	0.0046	0.1100
PYGM	0.0505	0.0828	0.0023	0.0669
ALDOA	0.0177	0.0299	0.0088	0.0237

^a^ Glucose uptake stimulating peptide (GUSP).

**Table 4:** DPP-IV inhibitory and stimulating the absorption of glucose bioactive peptide sequences released from the pork meat protein sequences after in silico digestion by pepsin, trypsin, and chymotrypsin. ^a^

Protein	Activity of Peptides	
	DPP-IV Inhibitory	Stimulating ^c^
ACTS	**EK**^(3) b^ [121-122][222-223][245-246]; **AL** [176-177]; **SL** [147-148]; **GY** [205-206]; **IL** [200-201]; **IW** [89-90]; **MK** [198-199]; **SF** [207-208]; **TL** [70-71]; **VK** [19-20]	**IL** [200-201]
MYH2	**EK**^(10)^ [280-281][450-451][915-916][1003-1004][1031-1032] [1262-1263][1264-1265][1290-1291][1465-1466][1581-1582]; **AL** ^(4)^ [413-414][1034-1035][1408-1409][1868-1869]; **SL** ^(2)^[599-600][1528-1529]; **GL** ^(2)^ [799-800][1903-1904]; **VR** ^(2)^ [696-697] [1883-1884]; **PL** [870-871]; **AF** ^(2)^ [641-642][850-851]; **GF** ^(3)^ [353-354][734-735][824-825]; **HW** [856-857]; **IL** ^(3)^ [739-740][1504-1505][1614-1615]; **IR** [259-260]; **MF** [452-453]; **MK** ^(3)^ [860-861][1536-1537][1844-1845]; **ML** ^(2)^ [170-171][1479-1480]; **MR** [1216-1217]; **NF** [1500-1501]; **NL** ^(3)^ [107-108][1013-1014][1558-1559]; **NY** [1700-1701]; **PF** ^(2)^ [31-32][324-325]; **PK** ^(2)^ [562-563][587-588]; **PW** [858-859]; **QF** [168-169]; **QL** ^(4)^ [287-288][920-921][1336-1337][1366-1367]; **QY** [471-472]; **SK** ^(3)^ [1307-1308][1410-1411][1960-1961]; **SY** ^(2)^ [293-294][1943-1944]; **TF** [528-529]; **TK** ^(7)^ [891-892][945-946][1029-1030][1059-1060][1063-1064][1421-1422][1467-1468]; **TL** ^(3)^ [639-640] [996-997] [1295-1296]; **TY** ^(2)^ [119-120][1915-1916]; **VF** [791-792]; **VK** ^(7)^ [48-49][419-420][1011-1012][1257-1258][1911-1912][1941-1942][1990-1991]; **VL** ^(3)^ [514-515][715-716][752-753]	**IL**^(4)^ [739-740] [1504-1505] [1614-1615] [1927-1928]; **VL** ^(3)^ [514-515] [715-716][752-753]
TPM3	**EK** [258-259]; **AL** [138-139]; **DR** ^(2)^ [20-21] [102-103]; **IL** [233-234]; **NR** [91-92]; **QL** [38-39]; **SK** [36-37]	**IL** [233-234]
TNNC2	**DR** [105-106]; **ML** [45-46]; **SY** [9-10]	-
TNNT3	**AL** [163-164]; **PL** [204-205]; **EY** [235-236]; **IR** [126-127]; **PK** [54-55]; **QL** [228-229]; **VL** [197-198]	**VL** [197-198]
TNNT1	**EK** [211-212]; **PL** [198-199]; **IL** [191-192]; **MR** [137-138]; **PK** [40-41]; **VK** [170-171]; **VL** [157-158]	**VL** [157-158]; **IL** [191-192]
TTN	**SL**^(2)^ [412-413][445-446]; **PL** [500-501]; **NF** [129-130]; **NY** [290-291]; **PK** [486-487]; **SW** ^(3)^ [74-75][377-378][576-577]; **SY** [302-303]; **TK** ^(2)^ [379-380][493-494]; **TL** ^(2)^ [233-234][235-236]; **VK** [410-411]; **VL** [220-221]	**VL** [220-221]
NEB	**VPL** [485-487]; **HL** ^(3)^ [610-611][1112-1113][1520-1521]; **EK** ^(4)^ [532-533] [569-570][603-604][1067-1068]; **AL** ^(2)^ [1435-1436][1744-1745]; **SL** ^(2)^ [155-156][1078-1079]; **GL** ^(4)^[659-660][910-911][1654-1655][1706-1707]; **VR** [78-79]; **PL** ^(2)^ [1140-1141][1746-1747]; **AW** [671-672]; **AF** [793-794]; **AY** ^(2)^ [657-658][908-909]; **DR** [1571-1572]; **EY** [848-849]; **GF** ^(3)^ [601-602] [784-785][852-853]; **HF** [1589-1590]; **HR** [1500-1501]; **HW** [102-103]; **IR** [1261-1262]; **MK** ^(6)^ [294-295][350-351][673-674][924-925][1069-1070][1587-1588]; **MR** [1175-1176]; **NL** ^(4)^ [75-76][687-688][1281-1282] [1677-1678]; **NY** ^(3)^ [469-470][537-538][702-703]; **PF** [1295-1296]; **PY** [52-53]; **QY** ^(2)^ [665-666][1350-1351]; **SK** ^(3)^ [107-108][605-606][1029-1030]; **TF** [1173-1174]; **TL** ^(4)^ [442-443][539-540][651-652][1503-1504]; **TR** ^(2)^ [1390-1391][1679-1680]; **VK** ^(2)^ [548-549][1318-1319]; **VL** ^(2)^ [366-367][575-576]; **VY** [1525-1526]	**VL**^(2)^ [366-367] [575-576]
MB	**HL** [49-50]; **EK** [42-43]; **AL** [139-140]; **GF** [155-156]; **IR** [31-32]; **QL** [9-10]; **VL** [11-12]	**VL** [11-12]
CKMT1	**AL** [19-20]; **SL** [178-179]; **GL** ^(2)^ [176-177][297-298]; **VR** [168-169]; **PL** [343-344]; **MW**[315-316]; **DR** [385-386]; **GY** [321-322]; **IL** ^(2)^ [323-324][354-355]; **IR** [153-154]; **IW** [268-269]; **PK** [352-353]; **QY** [46-47]; **SF** [265-266]; **SK** [346-347]; **TL** [87-88]; **VF** [290-291]; **VL** [163-164]	**VL** [163-164]; **IL** ^(2)^[323-324] [354-355]
CKM	**AL** [33-34]; **EK** [380-381]; **VR** [135-136]; **PL** [179-180]; **MW** [280-281]; **GY** ^(2)^ [143-144][286-287]; **NF** [13-14]; **SF** [230-231]; **SK** ^(2)^ [24-25][313-314]; **TL** ^(3)^ [35-36][52-53][145-146]; **TR** [323-324]; **VL** ^(2)^ [130-131][288-289], **VW** [233-234]	**VL**^(2)^ [130-131] [288-289]
GAPDH	**EK** [256-257]; **GY** ^(2)^ [281-282][327-328]; **HY** [39-40]; **MF** [44-45]; **QY** [46-47]; **VK** [316-317]	-
LDHA	**GL** [287-288]; **GY** [254-255]; **IL** [195-196]; **MK** [41-42]; **NL** ^(3)^ [110-111] [219-220][274-275]; **NR** [160-161]; **PK** [158-159]; **QF** [341-342]; **TY** [148-149]	**IL** [195-196]
PGK1	**AL**^(2)^ [206-207][364-365]; **SL** ^(2)^ [79-80][89-90]; **EW** [354-355]; **MK** [196-197]; **NY** [201-202]; **PF** [213-214]; **PK** [188-189]; **SK** [159-160]; **TF** [251-252]; ****TL**** [8-9]; **VK** [144-145]; **VL** ^(2)^ [255-256][419-420]	**VL**^(2)^ [255-256] [419-420]
PYGM	**HL**^(2)^ [35-36][412-413]; **EK** ^(3)^ [79-80][197-198][375-376]; **AL** ^(2)^ [55-56][106-107]; **SL** [89-90]; **GL** ^(3)^ [18-19][119-120] [156-157]; **PL** [4-5]; **AW** [377-378]; **AY** [57-58]; **DG** ^(3)^ [43-44][277-278][758-759]; **EW** [823-824]; **EY** [571-572]; **GY** ^(2)^ [161-162][750-751]; **HF** [37-38]; **IL** [363-364]; **IR** ^(2)^ [71-72] [548-549]; **ML** [705-706]; **MR** [361-362]; **NF** [31-32]; **NL** ^(2)^ [262-263][279-280]; **NR** ^(3)^ [33-34][425-426][607-608]; **QL** ^(2)^ [117-118][595-596]; **SY** [540-541]; **TL** ^(3)^ [39-40][97-98][204-205]; **TR** [825-826]; **VF** [798-799]; **VK** ^(2)^ [41-42][554-555]; **VL** [287-288]	**VL** [287-288]; **IL** [363-364]
**AL**DOA	**AL** [100-101]; **VL** [93-94]	**VL** [93-94]

^a^ Peptide sequences are listed using the single letter code for amino acids, ^b^ numbers in brackets refer to the total amount of specified active fragments, ^c^ GUSP.

**Table 5:** The results of the analysis of the physico-chemical properties of selected antidiabetic peptides.

Peptide	Molecular Weight (gmol^−1^)	Isoelectric Point	Net charge	Solubility ^a^	Bioactivity Score ^b^	Peptide	Molecular Weight (gmol^−1^)	Isoelectric Point	Net Charge	Solubility ^a^	Bioactivity Score ^b^
**AF**	236.27	3.77	0	-	0.97	**NR**	288.30	10.42	1	+	0.26
**AL**	202.25	3.70	0	-	0.44	**NY**	295.29	3.24	0	-	0.22
**AW**	275.30	3.66	0	-	0.97	**PL**	228.29	4.08	0	-	0.81
**AY**	252.27	3.66	0	-	0.35	**PF**	262.30	4.15	0	-	0.99
**DG**	190.15	0.68	−1	+	0.39	**PK**	243.30	10.57	1	+	0.33
**DR**	289.29	6.68	0	+	0.29	**PW**	301.34	4.04	0	-	0.99
**EK**	275.30	6.85	0	+	0.02	**PY**	278.30	3.85	0	-	0.74
**EW**	333.34	0.88	−1	+	0.59	**SF**	252.27	3.43	0	-	0.95
**EY**	310.30	0.95	−1	+	0.07	**SK**	233.27	9.86	1	+	0.07
**GF**	222.24	3.70	0	-	0.99	**SL**	218.25	3.37	0	-	0.33
**GL**	188.22	3.63	0	-	0.81	**SY**	268.27	3.39	0	-	0.26
**GY**	238.24	3.61	0	-	0.74	**SW**	219.30	3.34	0	-	0.93
**HF**	302.33	7.56	0	-	0.95	**TF**	266.29	3.36	0	-	0.83
**HL**	268.31	7.56	0.1	-	0.37	**TK**	247.29	9.28	1	+	0.03
**HR**	311.34	10.59	1.1	+	0.33	**TL**	232.28	3.32	0	-	0.14
**HW**	341.36	7.56	0.1	-	0.95	**TR**	275.31	10.53	1	+	0.13
**HY**	318.33	7.54	0.1	-	0.30	**TY**	282.29	3.35	0	-	0.11
**IL**	244.33	3.64	0	-	0.39	**QF**	293.32	3.41	0	-	0.95
**IR**	287.36	10.85	1	+	0.33	**QL**	259.30	3.34	0	-	0.29
**IW**	317.38	3.61	0	-	0.94	**QY**	309.32	3.36	0	-	0.23
**MF**	296.39	3.45	0	-	1.00	**VF**	264.32	3.67	0	-	0.82
**MK**	277.39	9.88	1	+	0.45	**VK**	245.32	10.10	1	+	0.03
**ML**	262.37	3.38	0	-	0.89	**VL**	230.30	3.60	0	-	0.13
**MR**	305.40	10.59	1	+	0.85	**VR**	273.33	10.10	1	+	0.11
**MW**	335.42	3.35	0	-	1.00	**VW**	303.36	3.57	0	-	0.80
**NF**	279.29	3.28	0	-	0.94	**VY**	280.32	3.59	0	-	0.10
**NL**	245.28	3.21	0	-	0.29	**VPL**	327.42	3.60	0	-	0.37

^a^ Estimated solubility in water where “+” means good solubility and “-” means poor solubility; ^b^ bioactivity score obtained with PeptideRanker tools.

**Table 6:** Highest occupied and lowest unoccupied molecular orbitals (HOMO and LUMO), their orbital energies, as well as the HOMO–LUMO energy gap for selected peptides from *Sus scrofa*.

Dipeptide	HOMO, *E* (eV)	LUMO, *E* (eV)	∆*E*_HL_ (eV)
GF 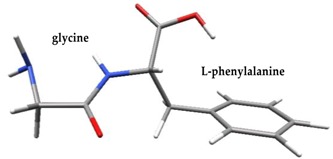	−6.97 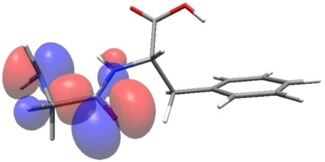	−1.04 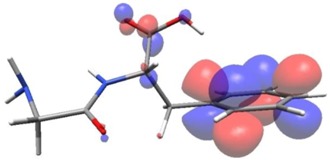	5.93
MW 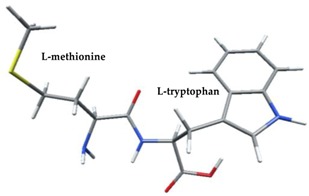	−6.03 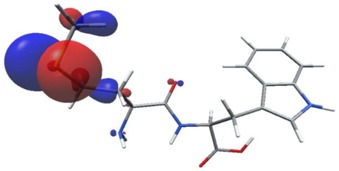	−1.09 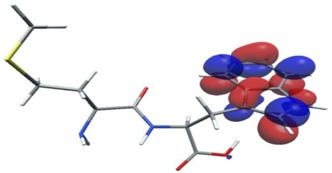	4.94
MF 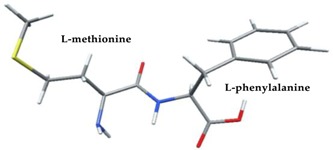	−6.06 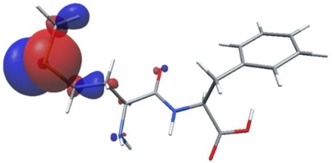	−1.20 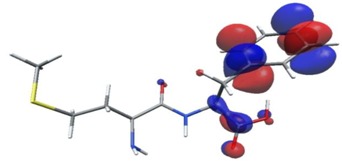	4.86
PP 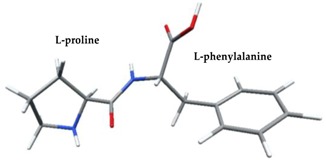	−6.35 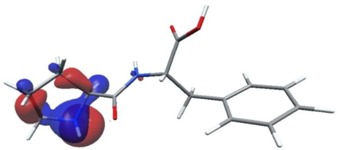	−0.85 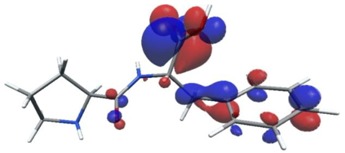	5.50
PW 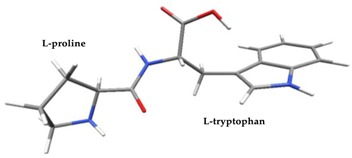	−6.23 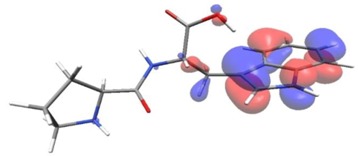	−1.16 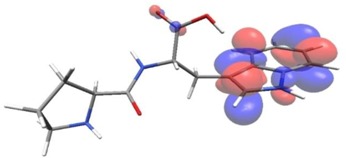	5.07
